# Prevalence and Risk Factors of Osteoporosis: A Cross-Sectional Study in a Tertiary Center

**DOI:** 10.3390/medicina60122109

**Published:** 2024-12-23

**Authors:** Samican Özmen, Sefa Kurt, Hikmet Tunç Timur, Onur Yavuz, Hakan Kula, Ayşegül Yılmaz Demir, Ali Balcı

**Affiliations:** 1 Department of Gynecology and Obstetrics, Torbalı State Hospital, İzmir 35860, Türkiye; samicanozmen@hotmail.com; 2 Department of Gynecology and Obstetrics, School of Medicine, Dokuz Eylül University, İzmir 35330, Türkiye; sefakurt@gmail.com (S.K.); o-yavuz@hotmail.com (O.Y.); hkula95@gmail.com (H.K.); dr.aysegulyilmaz@gmail.com (A.Y.D.); 3 Department of Radiology, School of Medicine, Dokuz Eylül University, İzmir 35330, Türkiye; ali.balci@deu.edu.tr

**Keywords:** osteoporosis, bone fractures, diabetes

## Abstract

*Background and Objectives:* Osteoporosis is a common disease of the skeletal system that increases the risk of fracture. The prevalence of osteoporosis has been increasing as the aging population increases, affecting more than 200 million people worldwide. This study aimed to shed light on the clinical impact of osteoporosis on women’s health and quality of life by evaluating the prevalence and risk factors for this disease among postmenopausal women, using a 10-year dataset from a tertiary center. *Materials and Methods:* This retrospective cohort study was conducted at Dokuz Eylül University, Department of Obstetrics and Gynecology, between 2010 and 2022. A total of 3289 postmenopausal women aged 50–60 years who were undergoing routine gynecological checkups were included in the study. Patients with a prior diagnosis, a history of medical conditions, or who were taking medications affecting bone mineral density (BMD) were excluded. Data on demographics, smoking status, diabetes status, body mass index (BMI), parity, and fracture history were collected. BMD scores were classified as normal, osteopenia, or osteoporosis. *Results:* The prevalence of osteoporosis was 10.4%. The median age of the patients was 56.0 years. Smoking, diabetes, and a history of bone fractures were found to significantly increase the risk of osteoporosis. BMI was found to have a protective effect against osteoporosis. According to multivariate analysis, the risk of osteoporosis increased by 2.46 times in patients who smoke, 3.78 times in patients with diabetes, and 6.23 times in patients with a history of fractures. *Conclusions:* Awareness of modifiable risk factors such as smoking is crucial for preventing osteoporosis-related complications. Diabetes, even when it is not complicated, increases the risk of osteoporosis. Physical exercise, calcium, and vitamin D intake are important, especially during young adulthood, as they are the primary contributors to peak bone mass. Because neck fractures are more common in patients with osteopenia, early diagnosis and routine screening can mitigate future complications.

## 1. Introduction

Osteoporosis is a systemic disease of the skeletal system, characterized by decreased bone mass and deterioration of bone microarchitecture, and is associated with increased bone fragility and risk of fracture [1]. Osteoporosis is a severe and progressive disease that remains silent until symptoms appear and stands out as the most common metabolic bone disease [2]. Pain, disability, reduced quality of life, and shortened life expectancy are the outcomes of osteoporosis [3].

Today, it is thought that 200 million people worldwide suffer from osteoporosis [2]. As a chronic and progressive skeletal disorder, the incidence of osteoporosis is rising in parallel with the aging population worldwide.

Although osteoporosis affects individuals of all ages and races, postmenopausal women constitute the highest-risk group for osteoporosis [4] because of the decreased estrogen levels [5]. Insufficient estrogen leads to increased osteoclast activity, resulting in bone trabecular weakening and an increased risk of fractures [6,7]. The increasing proportion of the elderly population, which results in prolonged durations of menopause, contributes to the increased frequency of osteoporosis and fractures [8].

Osteoporosis affects 10% of the world’s population and 30% of postmenopausal women [9]. Epidemiological studies estimate that approximately 10.2 million individuals over the age of 50 in the United States are affected by osteoporosis. This number is projected to increase by over 30% by 2030, yet it is still considered an underestimation by experts due to the high rates of underdiagnosis [10]. Hence, osteoporosis is defined by the WHO as a “global health problem” and the silent epidemic of the 21st century due to its impact on public health [11].

The most common and significant complication of osteoporosis is bone fracture. Currently, an estimated 2 million new osteoporotic fractures are diagnosed annually, a figure exceeding the annual incidence of myocardial infarction, breast cancer, and prostate cancer [12]. These fractures can be asymptomatic but may also lead to symptoms such as pain, loss of height, limitations in daily activities, decreased quality of life, and even death [2,13]. Osteoporotic fractures also increase the risk of subsequent bone fractures, contributing to the increased early mortality associated with osteoporosis. In 10% of cases, a new fracture occurs within the first year, 18% within the first two years, and 31% within the first five years following an initial osteoporotic fracture [14]. The mortality rate within the first year following an osteoporotic fracture is estimated to be 20%, and the risk of mortality increases 3 to 4 times over five years following these fractures [10].

Measuring the bone mineral density (BMD) by dual-energy X-ray absorptiometry (DXA) is the most commonly used method for diagnosing osteoporosis [15]. By measuring the transmission of X-ray photon beams through the spine and hip bones of the patient’s body, DXA allows for the evaluation of the bone’s calcium content and, consequently, its mineral density [2].

The standard deviation (SD) of BMD in individuals compared to young adults is referred to as the t score. Based on this score, individuals are classified as normal (t score > −1), osteopenic (−2.5 < t score < −1.0), or osteoporotic (t score ≤ −2.5) [3]. A decrease in BMD is a fundamental risk factor for developing osteoporosis, whereas many modifiable and nonmodifiable risk factors contribute to the progression of the disease. These factors are shown in Table 1 [16].

In addition to DXA, various technologies can evaluate BMD, bone structure, strength, and fracture risk. These methods include quantitative computed tomography (QCT) and quantitative ultrasound (QUS), as well as pulse echo ultrasound (PEUS) and peripheral QCT (pQCT), which are still in the clinical research phase, and magnetic resonance imaging (MRI). Additionally, although not approved for the diagnosis of osteoporosis, bone turnover markers such as osteocalcin, bone-specific alkaline phosphatase, and serum C-telopeptide may also be useful in diagnosing osteoporosis and assessing fracture risk [12].

The prevalence of osteoporosis and related fractures varies between countries, making it essential to evaluate the prevalence and risk factors individually within each country. Therefore, in this study, we aimed to investigate the prevalence of osteoporosis among postmenopausal women aged 50–60 years, using 10 years of data from a tertiary center, and to compare the findings with the existing literature. Our secondary objective was to assess the effects of age, body mass index (BMI), smoking, diabetes, parity, and fracture history on BMD and osteoporosis through multiple linear regression analysis in order to provide a comprehensive understanding of osteoporosis in our region.

## 2. Materials and Methods

### 2.1. Ethics Approval

Prior to the collection of data, this study was approved by the Non-Interventional Health Research Ethics Committee of Dokuz Eylül University (Protocol no: 2023/40-16). The study was conducted in accordance with the Declaration of Helsinki.

### 2.2. Participants and Study Design

**Figure 1:**
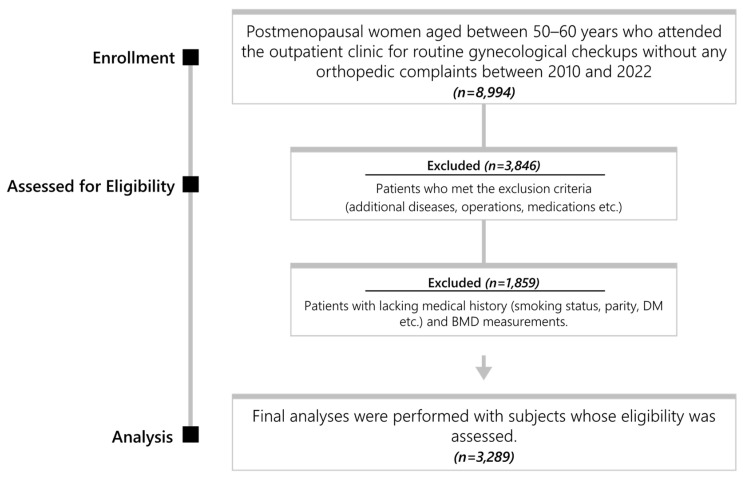
Flowchart.

This retrospective cohort was conducted at the Department of Obstetrics and Gynecology in Dokuz Eylül University Faculty of Medicine. All postmenopausal women aged between 50–60 years who attended the outpatient clinic for routine gynecological checkups without any orthopedic complaints and who underwent DXA scans for control purposes were included in this study. Data were collected over 10 years: from 2010 to 2022. Between the specified dates, 3289 out of the 8994 postmenopausal women aged 50–60 years, who attended the outpatient clinic for routine gynecological checkups without any orthopedic complaints, were included in the study (Figure 1).

The medical history of the subjects, including age, parity, BMI, smoking status, presence of diabetes, and history of low-trauma fractures occurring during the premenopausal period, was recorded. The prevalence of osteoporosis in the study population, factors affecting the risk of osteoporosis, and the statistical significance of these factors were calculated.

### 2.3. Exclusion Criteria

The following were excluded from the study: patients who were previously diagnosed with osteoporosis, those with a history of treatment for osteoporosis at any time in their lives; premenopausal patients within the specified age group; patients experiencing menopause before the age of 40 (premature menopause); patients with a history of hysterectomy and/or unilateral or bilateral salpingo-oophorectomy; patients with additional diseases that may cause low BMD such as rheumatoid arthritis, inflammatory bowel disease, celiac disease, any stage of renal insufficiency, sickle cell anemia, thyroid, parathyroid, and adrenal diseases; patients with a history of conditions increasing the risk of falls such as Parkinson’s disease, dementia, visual impairments, and vertigo; patients with a history of malignancies, especially breast cancer; patients receiving medical treatments that may affect bone metabolism such as androgen antagonists, aromatase inhibitors, proton pump inhibitors, SSRIs, thiazolidinediones, and anticonvulsants; patients receiving hormone replacement therapy; and patients for whom all requested data were not available.

### 2.4. BMD Measurement

The diagnosis of osteoporosis relied on BMD measurements. The BMD was calculated by dividing the total bone mineral content (g) by the surface area (cm^2^). BMD values were measured using the Primus DXA full-body fan-beam system (OsteoSys, Seoul, Korea). The PRIMUS system determines BMD values by analyzing images obtained from spine, femur, and forearm scans using fan beam technology. All BMD measurements were conducted by trained technicians. Daily calibrations were carried out on the measuring devices as per the instructions for use.

### 2.5. Definitions

Menopause was defined as the absence of uninterrupted menstruation for 12 months without any other physiological/pathological reasons or clinical intervention.

BMD measurements were evaluated according to WHO criteria. According to the BMD scores of the femoral neck, the subjects were divided into three groups: normal (t score > −1 SD), osteopenia (−2.5 < t score < −1.0), and osteoporosis (t score ≤ −2.5 SD) [12].

### 2.6. Statistical Analysis

Statistical analysis was performed using SPSS version 23.0 (SPSS, Inc., Chicago, IL, USA). Descriptive data for categorical variables were presented in tables with counts and percentages. The normal distribution of continuous variables was assessed visually (histograms and probability plots) and analytically (Kolmogorov–Smirnov test). Continuous variables that did not follow a normal distribution were presented as median (interquartile range). For comparisons between three independent groups with continuous variables that did not follow a normal distribution, the Kruskal–Wallis test was used, whereas the Mann–Whitney U test was used for comparisons between two independent groups. The chi-square test was used for comparisons of categorical variables. To predict risk factors for osteoporosis, univariate logistic regression analysis was performed. Significant variables were included in the multivariate logistic regression model.

To ensure adequate statistical power, the sample size was calculated using G-Power 3.1 (Heinrich-Heine-Universitat Düsseldorf, North Rhine-Westphalia, Germany). For the chi-square test of independence, we aimed for a minimum power of 0.95 and an alpha level of 0.05. An estimated effect size of 0.5 (large effect) was used, along with 2 degrees of freedom (reflecting our diagnostic groups). Based on these parameters, a minimum sample size of 62 participants per diagnostic group (healthy, osteopenia, osteoporosis) was required.

A *p* value < 0.05 was considered to indicate statistical significance.

## 3. Results

A total of 3289 subjects who met the inclusion criteria were included in the study. Among these subjects, 1500 (45.6%) were found to be healthy, whereas osteopenia was observed in 1448 (44%) cases, and osteoporosis was detected in 341 (10.4%) cases. Osteoporosis was detected in 115 (8.7%) of the women aged 50–55 years and 226 (11.5%) of the women aged 55–60 years. Of the 3289 patients included in the study, 1204 (36.6%) were smokers, 719 (21.9%) had diabetes, and 519 (15.8%) had a history of low-trauma fractures during the premenopausal period (Table 2).

The median age of the subjects included in the study was 56.0 (52.0–57.0) years, the median BMI was 26.9 (24.0–30.4), the median DXA t score was −1.1 (−1.8–−0.3), and the median parity was 1.0 (1.0–2.0). The numerical data are presented in Table 3.

**Figure 2:**
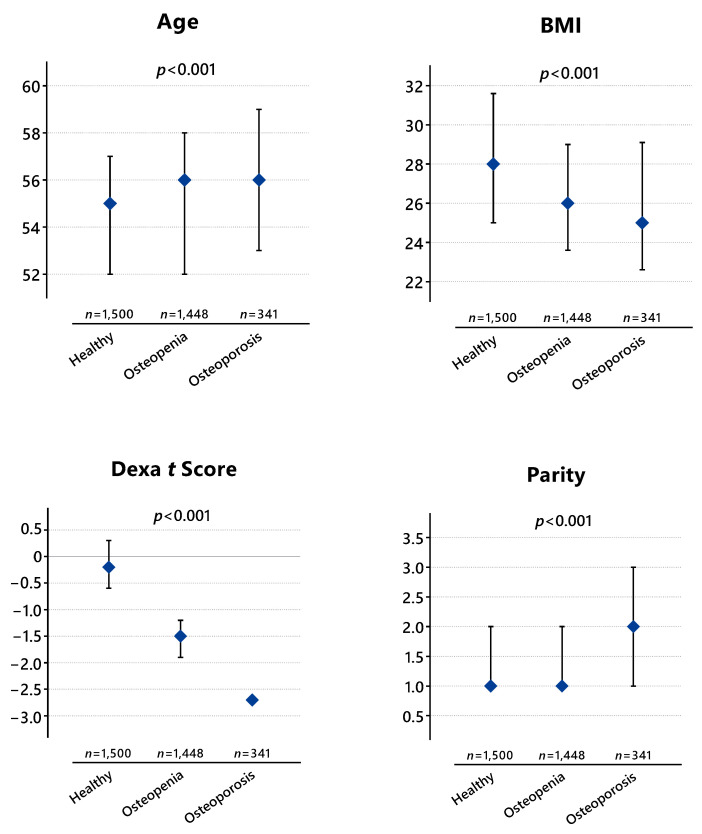
Comparison of Diagnostic Groups.

Figure 2 shows the comparison of diagnostic groups using numerical data. Accordingly, the median age of healthy subjects (patients without osteoporosis and osteopenia) is 55 (52–57) years, whereas the median age of cases with osteopenia is 56 (52–58 years), and that of cases with osteoporosis is 56 (53–59) years. A statistically significant difference in age is observed among the diagnostic groups (*p* < 0.001). The analysis revealed statistically significant differences between the healthy group and those with osteopenia (*p* = 0.002), between the healthy group and those with osteoporosis (*p* < 0.001), and between the group with osteopenia and the group with osteoporosis (*p* = 0.003).

The difference in BMI between the groups was also found to be statistically significant (*p* < 0.001). In the healthy group, the median BMI was 28 (25–31.6), whereas the median BMI of cases with osteopenia was 26 (23.6–29), and that of cases with osteoporosis was 25 (22.6–29.1). Accordingly, the differences between the healthy group and those with osteopenia (*p* < 0.001) and between the healthy group and those with osteoporosis (*p* < 0.001) were statistically significant, whereas no statistically significant difference was detected between the BMIs of cases with osteopenia and those with osteoporosis (*p* = 0.108).

The median DXA t score of healthy cases included in the study was −0.2 (−0.6–0.3), whereas the median t score of cases with osteopenia was −1.5 (−1.9–−1.2), and that of cases with osteoporosis was −2.7 (−2.9–−2.6). As expected, a statistically significant difference was detected among the groups in terms of the DXA t scores (*p* < 0.001).

In healthy cases, the median parity is 1 (1–2), whereas in cases with osteopenia, the median parity is 1 (1–2), and in cases with osteoporosis, the median parity was 2 (1–3). A statistically significant difference was observed among the diagnostic groups in terms of parity (*p* < 0.001). Although a statistically significant difference was found between the healthy group and those with osteoporosis (*p* < 0.001) and between cases with osteopenia and those with osteoporosis (*p* < 0.001), no statistically significant difference in parity was observed between cases with osteopenia and healthy cases (*p* = 0.736).

**Figure 3:**
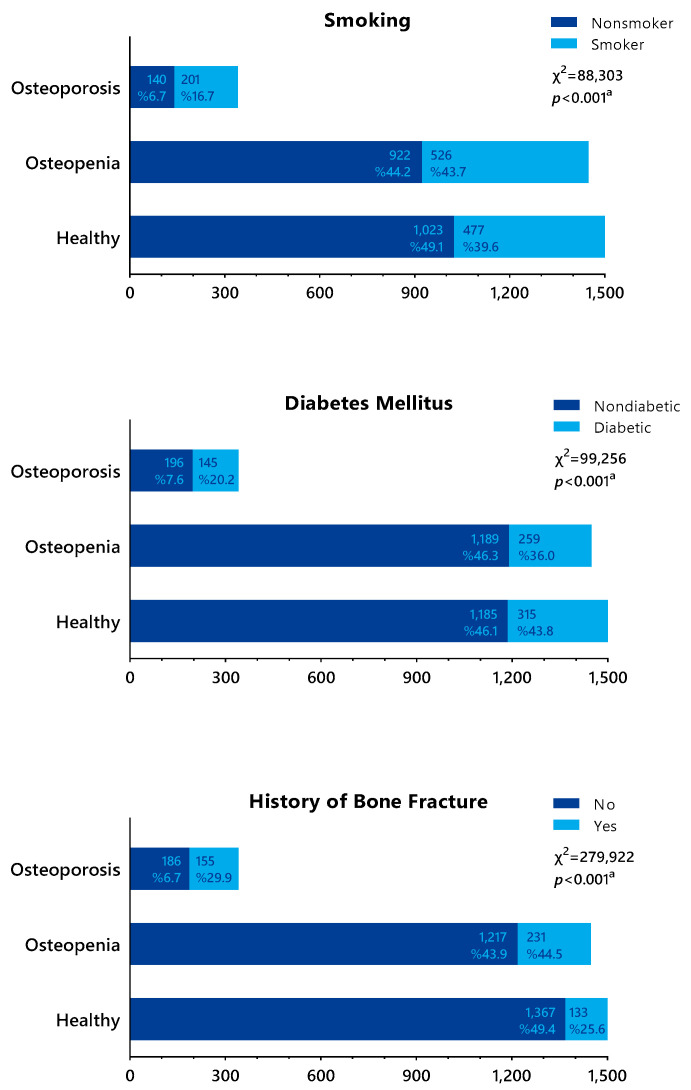
Comparison of diagnostic groups with risk factors. ^a^ chi-square test.

Figure 3 compares diagnostic groups with risk factors. The prevalence of osteoporosis among smokers was 16.7%, whereas the prevalence of osteopenia was 43.7%. Of the smokers, 39.6% did not have osteoporosis or osteopenia. Thus, a statistically significant difference was found between smoking status and the diagnosis groups (*p* < 0.001). According to our study, smoking significantly increases the risk of osteoporosis. The relationship between the presence of diabetes and the risk of osteoporosis was also statistically significant (*p* < 0.001). Of the patients with a history of fracture, 25.6% did not have osteopenia or osteoporosis, whereas 44.5% of these cases had osteopenia and 29.9% had osteoporosis. Therefore, a history of fracture significantly increases the risk of both osteopenia and osteoporosis.

The risk factors associated with osteoporosis according to the logistic regression model are shown in Table 4. Accordingly, in our study, the risk of osteoporosis increased by 2.46 times (95% CI: 1.92–3.15) in cases who smoked, 3.78 times (95% CI: 2.89–4.93) in cases who had diabetes, and 6.23 times (95% CI: 4.79–8.08) in cases with a history of fractures. An increase in BMI was identified as a protective factor against osteoporosis (0.91, 95% CI: 0.89–0.94). Compared to cases with a BMI above 30, our study revealed a 5.23-fold increase in osteoporosis risk in cases with a BMI below 18.5 (95% CI: 1.42–19.23). Osteoporosis risk was found to increase by 1.45 times (95% CI: 1.31–1.62) per parity.

When evaluated separately, each increase in age was associated with a 1.08-fold increase in the risk of osteoporosis; however, this difference did not reach statistical significance in the multivariate analysis.

## 4. Discussion

In this study, we investigated the prevalence of osteoporosis and the associated risk factors in postmenopausal women in our region by analyzing 10 years of data from a tertiary healthcare center. The key findings of our study include the increased risk of osteoporosis associated with smoking, diabetes, and a history of bone fractures in the premenopausal period, as well as the protective effect of an increase in BMI against osteoporosis. Notably, our study also found that the risk of osteoporosis was elevated even in cases of uncomplicated type 2 diabetes, which is a striking result.

Studies have reported the global prevalence of osteoporosis in postmenopausal women to be approximately 30% [1]. In our country, the estimated prevalence of osteoporosis among postmenopausal women is 27.2% [11]. In contrast, our study found the prevalence of osteoporosis to be 10.4%. This frequency was lower than that reported in the literature, likely because the individuals included in our study were younger postmenopausal women (max. age 60).

Poçan et al. [17] evaluated the prevalence of osteoporosis in 537 women aged 45 years and older, finding osteoporosis in 11% of cases. In their study, Hernlund et al. [18] calculated the prevalence of osteoporosis in individuals aged 50 and older to be 22%. The prevalence was 6.3% among women aged 50–54 years and 9.6% among women aged 55–59 years. Similarly, in our study, the prevalence of osteoporosis among women aged 50–55 years and 55–60 years was 8.7% and 11.5%, respectively.

Each 10-year increase in age increases the risk of osteoporosis by 1.4–1.8 times. This is attributed not only to hormonal decline but also to a decrease in bone wall thickness and an increase in bone resorption with age [19]. Although in our study age was not found to significantly increase the risk of osteoporosis according to the logistic regression model, when evaluated independently, each increase in age was associated with a 1.08-fold increase in osteoporosis risk. The reason for this discrepancy may be the inclusion of younger individuals in the study population after the regression model.

Cigarette smoking increases the risk of osteoporosis by generating superoxide radicals [20], reducing intestinal calcium absorption, enhancing osteoclast activity by suppressing osteoprotegerin production [2,21], and creating an antiestrogenic effect by decreasing aromatase activity [2,20]. Literature data show nearly twice the risk of osteoporosis in smokers compared to nonsmokers. In a previous study, the prevalence of osteoporosis was 31.3% among active smokers, 7.5% among nonsmokers, and 28.6% among former smokers [9]. In our study, the prevalence of osteoporosis among smokers and nonsmokers was 16.7% and 6.7%, respectively; thus, smoking was found to increase the risk of osteoporosis by 2.46 times. Therefore, our study also revealed a statistically significant association between smoking and osteoporosis, similar to the findings of previous studies.

Diabetes is a common endocrine disorder. According to the latest reports from the International Diabetes Federation in 2021, the estimated number of individuals with diabetes reached 530 million worldwide, and the prevalence of diabetes among women was 10.2% [22]. The literature shows a significant relationship between diabetes and increased fracture risk; however, data evaluating the relationship between diabetes and osteoporosis are limited.

It is known that type 1 diabetes leads to decreased BMD [23,24]. Researchers have suggested, however, that type 2 diabetes could potentially be protective against osteoporosis due to the frequent occurrence of obesity, which leads to increased BMD in these patients [25,26,27]. Although Leidig-Bruckner et al. [28] reported that the prevalence of osteoporosis in individuals with type 2 diabetes was significantly lower than that in healthy controls, DeShields et al. [24] reported that the risk of osteoporosis was greater in postmenopausal women with type 2 diabetes compared to the control group. Some studies argue that the increased risk of osteoporosis in individuals with type 2 diabetes only occurs in women [29,30]; even one study reported that this risk increase is only evident in postmenopausal White women [31]. In our study, the risk of osteoporosis was found to increase by 3.78 times in individuals with diabetes, and this increase was statistically significant. The following facts contribute to an objective evaluation of the relationship between osteoporosis risk and type 2 diabetes: all participants in our study had type 2 diabetes, glycemic control was achieved in the cases, the mean age of the participants was lower compared to previous studies, and no diabetes-related complications were observed in any of the cases. Additionally, these data distinguish our study from previous research, as previous studies have not separately assessed complicated and uncomplicated diabetes cases.

Studies have shown that increased body weight is associated with increased BMD, which may be protective against osteoporosis [4,17,32]. During the postmenopausal period, estrogen secreted from androgen receptors in adipose tissue may contribute to the protective effect of increased BMI against osteoporosis [8]. It has also been shown that BMD is lower in underweight individuals [23,32,33]. Skrzek et al. [34] reported that the risk of osteoporosis is lowest in postmenopausal women with a BMI between 26 and 27.9 kg/m^2^. Lee et al. [35] reported that each 1 kg/m^2^ increase in BMI reduces the risk of osteoporosis by 13% in women. Similarly, we found that for each unit increase in BMI, the risk of osteoporosis decreased by 0.91. In our study, compared to women with a BMI over 30, women with a BMI below 18.5 were found to have a 5.23-fold increase in the risk of osteoporosis.

Fractures occurring at any stage of life are considered a risk factor for osteoporosis. Although the increase in bone mass begins in utero and continues until the age of 40, the primary contributor to this process is the acquisition of bone mass during adolescence [36]. Low-trauma fractures before menopause may be attributed to the reduced peak bone mass in patients. According to the SAPOS study, one of the major factors associated with low mineral density is a history of bone fractures before menopause [4]. According to Charde et al. [37], a history of previous fractures and experiencing falls more than twice are risk factors for bone fractures in later stages of life. Like in previous studies, we also found a significant relationship between a history of fractures and osteoporosis and showed a 6.23-fold increase in the risk of osteoporosis in the presence of low-intensity fractures. The high prevalence of a history of fractures observed in our study may be attributed to the fact that the study was conducted in a tertiary center that is a reference center for the treatment of bone fractures in the region. We obtained the data on the history of fractures solely from the verbal anamnesis of the patients, which may have also contributed to this greater number than usual.

According to our study, nearly one-third of the patients with a history of fractures already had osteoporosis (29.9%). One notable finding of our study is that despite the increased risk, patients with a history of fractures were not routinely monitored for osteoporosis. These data highlight the inadequacy of population-based osteoporosis screening, which is consistent with findings from studies conducted in our country. A study conducted in 2019 showed that 82% of patients who underwent hemiarthroplasty did not receive treatment for osteoporosis, even after a history of fracture [38]. Recently, a systematic review involving adults aged 50–89 years reported that more than 75% of high-risk individuals did not initiate appropriate treatment after screening [39]. In this context, expanding routine screening, especially for high-risk patients, will lead to a significant reduction in morbidity and mortality associated with osteoporosis.

We found osteopenia in 44% (1448 cases) of all cases. Given that an estimated 23.7% of patients with osteopenia will develop osteoporosis within 8.5 years [40], this high prevalence of osteopenia in the population is indeed concerning. Considering the asymptomatic nature of the disease until bone fractures occur, the importance of routine screening becomes evident once again.

The data regarding the relationship between parity and osteoporosis are conflicting. Various researchers have suggested that multiparity significantly increases BMD and thus may be protective against osteoporosis [41,42]. In a recent study by Peker et al. [43], it was shown that the prevalence of osteoporosis in women who had <5 births was significantly lower compared to those who had given birth more frequently. In many similar studies, multiparity has been shown to be a risk factor for osteoporosis [44,45]. We found that multiparity is a risk factor for osteoporosis, with each increase in parity being associated with a 1.45-fold increase in the risk of osteoporosis.

Our study has limitations and strengths. One of the strengths of our study is the exclusion of all cases with additional diseases or medication use that could affect BMD, except for uncomplicated type 2 diabetes cases. Furthermore, we also excluded patients with diabetes-related complications and those with conditions such as vertigo, Parkinson’s disease, and visual impairments, which increase the risk of falls. This approach allowed for the objective assessment of conditions that pose a risk for osteoporosis. Furthermore, the inclusion of cases from a relatively young age group ensures that any additional factors that may increase the risk of osteoporosis or fractures do not influence the study’s data. Because our study did not involve a questionnaire survey as in previous research, the diagnosis of osteoporosis was objectively determined based on DXA values rather than patients’ medical histories. The exclusion of individuals who had received treatment for osteoporosis at any point in their lives from the study also enabled the objective evaluation of risk factors and osteoporosis prevalence in our study. Another factor that makes our study valuable is the scarcity of recent studies with such a large number of cases regarding osteoporosis risk factors and the prevalence of the disease in our country, as there are significant differences in osteoporosis and fracture prevalence among countries.

Indeed, our study has several limitations. In particular, being conducted in a socioeconomically developed region of our country and being a single-center study may have led to a lower participation rate of individuals from rural areas. Additionally, the lack of assessment of participants’ dietary and exercise habits, as well as their vitamin D and calcium levels, is another limitation of the study. Relying solely on DXA for the diagnosis of osteoporosis without confirming the diagnosis with any other method can also be considered a limitation. The lack of evaluation for additional conditions such as osteomalacia and osteoarthritis may have resulted in overestimated osteoporosis rates. In our study, we demonstrated that smoking increases the risk of osteoporosis. However, we did not categorize the cases into subgroups of current smokers or former smokers. Additionally, we did not assess the number of cigarettes smoked per day in the smoking group. Although all diabetic cases in our study were non-complicated, we lacked data on the duration of the disease. Another limitation of our study is that the history of bone fractures in patients was based solely on verbal anamnesis. Bone fractures from previous years were not identified through medical records; instead, this information was verbally obtained from the patients and recorded. This may have led to a higher prevalence of bone fractures compared to the general population. These limitations should also be acknowledged in our study.

## 5. Conclusions

In our study, it has been demonstrated that decreased body weight, smoking, and type 2 diabetes significantly increase the risk of osteoporosis. Given that the risk of bone fractures remains higher, even after quitting smoking, compared to individuals who have never smoked, avoiding smoking altogether or quitting smoking will lead to a significant reduction in the risk of osteoporosis. Considering that a history of fractures increases the risk of osteoporosis, it is important for at-risk individuals to avoid a sedentary lifestyle, consume adequate amounts of calcium and vitamin D through diet, and take preventive measures by increasing peak bone mass through regular physical exercise and a balanced diet, especially during young adulthood, to prevent the disease. Given that osteopenia cases constitute the majority of the population and that there is no consensus on treatment for these cases, the early detection of these individuals and the prevention of potential complications through routine screening and close monitoring of at-risk individuals are of great importance for both public health and health care economics in countries.

Future studies conducted with a larger number of patients can demonstrate changes in the prevalence of osteoporosis over the years and the effects of increasing awareness, preventive measures, and the introduction of new diagnostic and treatment methods on its prevalence.

## Figures and Tables

**Table 1:** Risk factors for osteoporosis.

Gender
Age
Ethnicity
Family history
Lower body mass index
Hormone disorders
*Amenorrhea* *Early menopause/menopause* *Hypogonadism* *Thyroid/Parathyroid disorders* *Panhypopituitarism* *Hyperprolactinemia*
Calcium, protein, and vitamin D intake
Drugs
*Anticonvulsants* *Glucocorticoids* *GnRH agonists/antagonists* *SSRIs* *Thiazolidinediones* *Aromatase inhibitors*
Sedentary lifestyle
Smoking and alcohol intake
Comorbid diseases
*Hypercalciuria* *Osteogenesis imperfecta* *Cystic fibrosis* *Cushing Syndrome* *Celiac disease* *Inflammatory bowel diseases* *Diabetes mellitus*

**Table 2:** Descriptive characteristics of the subjects included in the study.

	Number (%)
Diagnosis (*n* = 3289)	
*Normal*	1500 (45.6)
*Osteopenia*	1448 (44.0)
*Osteoporosis*	341 (10.4)
Smoking (*n* = 3289)	
*Nonsmoker*	2085 (63.4)
*Smoker*	1204 (36.6)
Diabetes Mellitus (*n* = 3289)	
*Nondiabetic*	2570 (78.1)
*Diabetic*	719 (21.9)
History of bone fracture (*n* = 3289)	
*No*	2770 (84.2)
*Yes*	519 (15.8)

**Table 3:** Numerical characteristics of the subjects included in the study.

	Median (IQR)
Age (*n* = 3289)	56.0 (52.0–57.0)
BMI (*n* = 3289)	26.9 (24.0–30.4)
Dexa t (*n* = 3289)	−1.1 (−1.8–−0.3)
Parity (*n* = 3289)	1.0 (1.0–2.0)

IQR: interquartile range; BMI: body mass index.

**Table 4:** Risk factors associated with osteoporosis according to the logistic regression model.

Variable	Univariate Logistic Regression				Multivariate Logistic Regression			
	Wald	SE	OR (95% CI)	*p*	Wald	SE	OR (95% CI)	*p*
Age	17.33	0.019	1.08 (1.04–1.12)	**<0.001**	0.46	0.022	1.015 (0.97–1.05)	**0.496**
DM	88.31	0.119	3.06 (2.42–3.86)	**<0.001**	96.23	0.136	3.78 (2.89–4.93)	**<0.001**
BMI	24.94	0.13	0.93 (0.91–0.96)	**<0.001**	37.79	0.014	0.91 (0.89–0.94)	**<0.001**
Parity	56.49	0.048	1.43 (1.30–1.57)	**<0.001**	49.26	0.054	1.45 (1.31–1.62)	**<0.001**
Smoking	76.92	0.117	2.78 (2.21–3.50)	**<0.001**	50.92	0.126	2.46 (1.92–3.15)	**<0.001**
History of fracture	211.19	0.122	5.91 (4.65–7.51)	**<0.001**	188.95	0.133	6.23 (4.79–8.08)	**<0.001**

DM: diabetes mellitus; BMI: body mass index.

## Data Availability

The datasets generated and analyzed during the current study are available from the corresponding author upon reasonable request.
